# Physio-Biochemical and Agronomic Responses of Faba Beans to Exogenously Applied Nano-Silicon Under Drought Stress Conditions

**DOI:** 10.3389/fpls.2021.637783

**Published:** 2021-09-16

**Authors:** El-Sayed M. Desoky, Elsayed Mansour, El-Sayed E. A. El-Sobky, Mohamed I. Abdul-Hamid, Taha F. Taha, Hend A. Elakkad, Safaa M. A. I. Arnaout, Rania S. M. Eid, Khaled A. El-Tarabily, Mohamed A. T. Yasin

**Affiliations:** ^1^Botany Department, Faculty of Agriculture, Zagazig University, Zagazig, Egypt; ^2^Agronomy Department, Faculty of Agriculture, Zagazig University, Zagazig, Egypt; ^3^Biochemistry Department, Faculty of Agriculture, Zagazig University, Zagazig, Egypt; ^4^Agricultural Botany Department, Faculty of Agriculture, Benha University, Banha, Egypt; ^5^Department of Biology, College of Science, United Arab Emirates University, Al-Ain, United Arab Emirates; ^6^Harry Butler Institute, Murdoch University, Murdoch, WA, Australia

**Keywords:** antioxidants, crop water productivity, irrigation regimes, Mediterranean region, nano-SiO_2_, principal components analysis, yield contributing traits

## Abstract

Nano-silicon application is an efficient novel approach to mitigate the deleterious impacts of drought stress on field crops, which is expected to increase owing to climate change, especially in arid regions. Two-season field studies investigated the influence of foliar-applied nano-silicon (0.5, 1, and 1.5 mM) on physiological and biochemical attributes and their impacts on crop water productivity (CWP) and the agronomic traits of faba beans (*Vicia faba*). The plants were evaluated under two irrigation regimes: well-watered (100% ETc giving 406 mm ha^−1^) and drought stress (65% ETc giving 264 mm ha^−1^). It was found that drought stress significantly decreased gas exchange (leaf net photosynthetic rate, stomatal conductance, and rate of transpiration), water relations (relative water content and membrane stability index), nutrient uptake (N, P, K^+^, and Ca^+2^), flavonoids, and phenolic content. In contrast, drought stress significantly increased oxidative stress (H_2_O_2_ and ${\text{O}}_{2}^{\cdot -}$) and enzymatic and non-enzymatic antioxidant activities compared with the well-watered treatment. These influences of drought stress were negatively reflected in seed yield-related traits and CWP. However, foliar treatment with nano-silicon, particularly with 1.5 mM, limited the devastating impact of drought stress and markedly enhanced all the aforementioned parameters. Therefore, exogenously applied nano-silicon could be used to improve the CWP and seed and biological yields of faba bean plants under conditions with low water availability in arid environments.

## Introduction

Faba beans (*Vicia faba* L.) are an important legume crop grown worldwide (Gasim et al., 2015). These plants have high crude protein content and essential amino acids (Vogelsang-O'dwyer et al., 2020) and improve soil nitrogen content through the symbiotic fixation of atmospheric nitrogen, which reduces the requirement for nitrogen fertilizer in agricultural production systems (Liu et al., 2019). Faba beans are cultivated in the Mediterranean region as a rotational crop and fix more than 80% of the nitrogen requirements of the plant (Denton et al., 2017). However, it is highly sensitive to water deficits compared with other field crops (Parvin et al., 2019).

The Mediterranean region is one of the most vulnerable areas to the deleterious impacts of climate change, with fluctuations in precipitation and water shortage being projected to increase, particularly in arid and semi-arid environments (García-Ruiz et al., 2011; Cook et al., 2016; Mansour et al., 2018; Spinoni et al., 2018; Chiwetalu et al., 2020). As a result, water scarcity causes destructive alterations in the biochemical and physiological processes of plants and, consequently, reduces their growth and productivity (Siddiqui et al., 2015; Attia et al., 2021; Desoky et al., 2021; Mansour et al., 2021). Therefore, it is crucial to mitigate the deleterious impacts of water deficiency using practical approaches to boost drought tolerance in field crops (Semida et al., 2020; Abd El-Mageed et al., 2021; El-Sanatawy et al., 2021).

Silicon (Si) considerably increases mechanical strength and membrane stability of cell. It also maintains membrane integrity, mineral nutrition, photosynthesis efficiency, and the tolerance defense system (Spinoni et al., 2018; Desoky et al., 2020). As a result, Si can be used to alleviate the negative impacts of water deficits and improve plant growth and productivity owing to its beneficial physicomechanical functions (Rady et al., 2019).

Recently, nanomaterials have become a desirable solution to many technological and environmental challenges in numerous fields (Ansari and Husain, 2012). Nano-silicon has displayed superior physicochemical properties owing to its microscopic size compared with bulk Si (Prasad et al., 2012; Rastogi et al., 2019). Furthermore, nano-silicon has a larger surface area, greater surface reactivity and solubility, and numerous well-characterized surface properties compared with bulk Si (Qados and Moftah, 2015). In particular, particle size is considered to be one of the most crucial factors impacting particle adhesion, uptake, and transportation into plant cells (Smith et al., 2008; Wang et al., 2009). In addition, nanoparticles interact with plant cells and assist in the transportation of different substances that can regulate plant metabolism and several physiological processes (Galbraith, 2007; Torney et al., 2007; Giraldo et al., 2014).

Investigations into the influence of nano metals and their mechanisms are still at the rudimentary stage. Studies related to nano-silicon application and its contribution to the attenuation of the adverse impacts of drought stress and the increasing of faba bean productivity under water-deficit conditions, particularly under field conditions, are lacking. Based on the results of previous investigations, we hypothesized that the application of nano-silicon would notably improve faba bean plant performance (growth and productivity) by improving the efficiency of enzymatic and non-enzymatic antioxidants, in turn reducing the overproduction of reactive oxygen species (ROS). Accordingly, we investigated the role of exogenously applied nano-silicon dioxide (SiO_2_) at different concentrations in ameliorating the drought tolerance of faba bean plants at the morphological, physiological, biochemical, and agronomic levels. This knowledge will assist in enhancing the drought tolerance of faba bean plants for their cultivation in arid environments.

## Materials and Methods

### Description of the Experimental Site

A field experiment was undertaken during the 2018–2019 and 2019–2020 winter growing seasons at the experimental farm of the Faculty of Agriculture, Zagazig University, Zagazig, Egypt (30°36′57″N, 31°46′58″E). The site was characterized by low precipitation and an arid climate, with an average annual rainfall of ~60 mm. The results of the soil analysis, including bulk soil density, field capacity, wilting point, pH, texture, and soil composition, are presented in Supplementary Table 1. The monthly minimum and maximum temperatures and rainfall for the two winters and the 35-year averages (from 1986 to 2020) were obtained from a station close to the experimental site (Supplementary Table 2).

### Agronomic Practices

Phosphorus fertilizer was added at a rate of 31 kg P ha^−1^ as calcium superphosphate [Ca(H_2_PO_4_)_2_, 15.5% P_2_O_5_] before sowing. Nitrogen (N) fertilizer was added at a rate of 45 kg N ha^−1^ as ammonium-sulfate [(NH_4_)_2_SO_4_, 21% N] as fertigation one time at sowing. Potassium (K) fertilizer was applied at a rate of 95 kg K ha^−1^ as potassium sulfate (K_2_SO_4_, 48% K_2_O) in two equal doses every two weeks after sowing. The sowing dates of both seasons were performed according to the optimal period for growing faba beans in the region during the first week of November. The genotype used in this experiment was a recommended commercial cultivar in the region (Giza-843). Standard agronomic practices, comprising drip irrigation, sowing date, chemical fertilization, weed, disease, and pest control, were applied as recommended for the commercial production of faba beans.

### Experimental Design and Irrigation Regimes

The experimental design was a split-plot, with randomized irrigation regimes in the main plots and foliar treatments in sub-plots in three replicates. Each plot consisted of six rows that were 5 m long with 0.65 m between rows. The plant spacing was 0.15 m, resulting in ~205,130 plants per ha^−1^. A drip irrigation system was used to meet the study objectives, with drip laterals and emitters were spaced at 0.65 and 0.3 m, respectively. The operating pressure and emitter flow rate were kept at 1 bar and 4 L h^−1^, respectively, and maintained using a valve and pressure gauge for each irrigation sector. Irrigation water quantity was measured independently for each irrigation regime using a flow meter. Irrigation scheduling was based on potential crop evapotranspiration (ETc) replacement according to the crop coefficient approach (Allen et al., 1998). The ETc was determined by multiplying the daily reference evapotranspiration (ET_o_) by the Food and Agriculture Organization (FAO) crop coefficients (Kc) of faba beans (Allen et al., 1998). The ET_o_ was determined from weather data using the FAO-56-standardized Penman–Monteith equation as stated by Allen et al. (1998). Daily meteorological data, including minimum, maximum, and dew point temperatures and wind speed, were taken from the closest weather station to calculate the ET_o_. The Kc figures for faba beans, as suggested by the FAO-56, were altered based on the obtained climatic values, including the wind speed and relative humidity of the experimental site. During the first and second growing seasons, the total amount of the full irrigation regime (100% ETc) was 400 and 412 mm ha^−1^, respectively. The drought stress regime was 35% less (260 and 268 mm ha^−1^) than the well-watered treatment from the seedling establishment to physiological maturity in both seasons. Irrigation was applied weekly from full emergence to flowering and then two times a week from flowering to maturity. Irrigation was discontinued 2 weeks before harvest (mid-April).

### Foliar Application of Nano-SiO_2_

Nano-silicon dioxide (99.5% pure; 20–30 nm) (Sigma-Aldrich Chemie GmbH, Taufkirchen, Germany), with a corresponding surface area of 180–600 m^2^ g^−1^, was used in the study (Supplementary Figure 1). Foliar sprays of 0, 0.5, 1, and 1.5 mM nano-SiO_2_ were applied using a pressurized spray bottle with 0.1% Tween 20 as a surface spreader. Spraying with distilled water was used as a control for foliar treatments.

### Agronomic Traits Measurements

At the end of the growing season, the plant height, which was from the soil surface to the uppermost leaf tip, was measured for 10 replicate plants in each plot. Three middle rows from each plot were harvested from a total area of 9.8 m^2^ to determine the yield components (number of pods per plant and seeds per pod and 100-seed weight), seed yield, and aboveground biomass. The 100-seed weight was assessed from the weight of the three sets of 100 seeds.

### Crop Water Productivity

The crop water productivity (kg m^−3^) for seed yield (CWP_s_) and aboveground biomass (CWP_ab_) was estimated as the ratio of seed yield or aboveground biomass (kg ha^−1^) to crop evapotranspiration (ET, m^3^) following the formula of Pereira et al. (2012) and Fernández et al. (2020):


$$\begin{array}{l} CWP\;\left(kg\;m^{-3}\right)=\frac{Yield\;}{ET} \end{array}$$

Crop evapotranspiration (ET, mm) was calculated according to the water balance equation (James, 1988): *ET* = *IW* + *P* + *Cr* + *Dp* ± *Rf* ± Δ*S*, where IW is the irrigation water amount (mm), P is the seasonal precipitation (mm), Cr is the capillary rise to the root zone (mm), Dp is the deep percolation (mm), Rf is the surface runoff (mm), and ΔS is the soil moisture change in the crop root zone (mm). The Cr in this study was zero as the groundwater table was 15 m below the ground surface. The Dp and Rf were neglected owing to the use of a drip irrigation system. Furthermore, the soil water content was determined using an oven drying method for all the experimental plots. Soil samples were collected at planting and harvest from soil depths of 0–30, 30–60, and 60–90 cm to estimate the initial and final soil moisture content during the two growing seasons. The figures were converted to a volumetric basis and multiplied by soil depth and bulk density.

### Determination of PSII Quantum Yield and CO_2_ Fixation Rate

All gas exchange measurements were performed using a Li-6400XT portable photosynthesis system equipped with a 6400-40 leaf chamber fluorescence head and a 6400-02 B LED light source (Li-Cor Inc., Lincoln, NE, USA) between 09:00 and 11:00 a.m. To avoid errors, CO_2_ leakage was corrected according to Flexas et al. (2007). All types of measurement (light response curves and CO_2_ response curves) were applied on the third leaves of three plants at each light intensity. Before starting the CO_2_ response curve measurements, the leaf was adapted for 5–20 min to ensure that photosynthesis, stomatal conductance, and fluorescence signal were stable and rubisco was fully activated. The first point was measured under a reference CO_2_ concentration (C_a_) of 400 ppm. The A–Ci measurements were conducted at the reference CO_2_ concentration of 400 ppm, followed by 300, 200, 100, 50, 150, 250, 350, 600, 900, 1,200, and 1,500 ppm under the saturating light conditions of 1,300 μmol m^2^ s^−1^ (Loriaux et al., 2013). The fluorometer measuring light was turned on and set to measure light frequency = 10 kHz, intensity = 3, filter = 5, and gain = 10. The flash was set to multiphase pulse, with a target intensity = 9, ramp depth = 30%, measuring frequency = 20 kHz, and filter = 50 kHz. The three phases were 320, 350, and 200 ms long (Chen et al., 2014).

### Determination of Relative Water Content, the Membrane Stability Index, Leaf Soluble Sugars, and Proline Content

Relative water content (RWC) was assessed following the method of Osman and Rady (2014) and determined using the following equation: $RWC\;\left(\%\right)=\frac{FM-DM\;}{TM-DM}\times 100$, where FM is fresh mass, DM is dry mass, and TM is turgid mass. The membrane stability index (MSI) was determined by adding 200 mg of fresh leaves into test tubes containing 10 cm^3^ of double-distilled water. One set was heated at 40°C for 30 min in a water bath, and the electrical conductivity of the solution was recorded on a conductivity bridge (C1). A replicated set was boiled at 100°C in a water bath for 10 min, with conductivity also being measured (C2). The MSI was calculated as described by Rady (2011) using the following equation: $MSI\;\left(\%\right)=1-\frac{EC_{1}\;}{EC_{2}}\times 100$, where EC_1_ is the electrical conductivity at 40°C and EC_2_ is the electrical conductivity at 100°C.

The total soluble sugars of 0.2 g of leaf washed with 5 ml of 70% ethanol and homogenized with 5 ml of 96% ethanol were determined following the method of Irigoyen et al. (1992). Briefly, the extract was centrifuged at 1,372 × *g* for 10 min, and the supernatant was collected and stored at 4°C. Freshly prepared anthrone (3 ml) was added to a 0.1 ml supernatant and incubated in a hot water bath for 10 min. Absorbance was measured at 520 nm (Jenway Spectrophotometer 6705, Staffordshire, UK), and the sugar content was determined from the standard curve (Supplementary Figure 2). One gram of glucose was dissolved in distilled water, with the volume amounting to 1 L. The different volumes of the glucose solution were taken and made to amount to 100 ml with distilled water in volumetric flasks. Finally, the relationship between the readings at 520 nm and the known concentration of glucose was plotted (Supplementary Figure 2). A rapid colorimetric assay was performed to measure the proline content in 0.5 g dried leaf samples according to Bates et al. (1973). The absorbance was recorded at 520 nm, with the proline content being determined from the standard curve. The proline standard curve was developed using 1 g of pure proline, which was purchased from Sigma-Aldrich (Supplementary Figure 3).

### Assessing Antioxidant Enzyme Activity

Enzyme extraction was performed according to the method of Vitória et al. (2001). Briefly, fresh leaf samples were collected in an icebox and taken to the laboratory. Distilled water was used to wash the leaves, following which, the surfaces of the leaves were wiped out of moisture. The leaf sample (0.5 g) was homogenized in 0.1 M of an ice-cold phosphate buffer (pH 7.5) containing 0.5 mM of ethylenediaminetetraacetic acid (EDTA) using a pre-chilled mortar and pestle. The homogenate was then transferred to centrifuge tubes and centrifuged at 4°C in a Beckman Coulter refrigerated centrifuge (Beckman Coulter, Inc., California, USA) at 15,000 × *g* for 15 min. The supernatant was transferred to 30-ml tubes and referred to the enzyme extract.

Catalase (CAT) was estimated spectrophotometrically according to Britton and Mehley (1955). Briefly, the enzyme extract (100 μl) was added to 100 μl of 100 mM of H_2_O_2_, and the total volume has amounted to 1 ml with 250 mM of the phosphate buffer at pH 6.8. The reduction in optical density at 240 nm against the blank was measured every minute. The activity of peroxidase (POD) was estimated as described by Thomas et al. (1982) using guaiacol as the substrate. The reaction mixture contained 3 ml of the phosphate buffer (0.1 M, pH 7), 50 ml of enzyme extract, 30 ml of H_2_O_2_ (20 mM), and 50 ml of guaiacol (20 mM). The reaction mixture was incubated in a cuvette for 10 min at room temperature. The optical density was recorded at 436 nm, and the enzyme activity was expressed as the number of absorbance units g^−1^ fresh weight of leaves. The superoxide dismutase (SOD) activity was determined by recording the reduction in the absorbance of the superoxide-nitro blue tetrazolium complex by the enzyme SOD (Sairam et al., 2002). Approximately 3 ml of a reaction mixture comprising 0.2 ml of 200 mM methionine, 0.1 ml of 3 mM EDTA, 0.1 ml of 1.5 M sodium carbonate, 1.5 ml of 100 mM potassium phosphate buffer pH 7, 0.1 ml of 2.25 mM nitro blue tetrazolium, 1 ml of distilled water, and 0.05 ml of the enzyme, which were all collected in duplicates from each of the enzyme samples into test tubes. Two tubes without the enzyme extract were used as controls. The reaction was started by adding 0.1 ml riboflavin (60 μM) and placing the tubes below a light source (two 15-W fluorescent lamps) for 15 min. The reaction was stopped by turning off the light and covering the tubes with a black cloth. The tubes without the enzyme developed the maximum color. A non-irradiated complete reaction mixture that did not develop color served as a blank. The absorbance was recorded at 560 nm, and one unit of enzyme activity was taken as the quantity of enzymes that reduced the absorbance readings of samples to 50% compared with the tubes lacking enzymes.

### Determination of Antioxidant Compounds and Oxidative Stress (H_2_O_2_ and ${\text{O}}_{2}^{\cdot -}$) Content

The content of ascorbate **(**AsA; μmol g^−1^ FW) was determined as described by Kampfenkel et al. (1995). Reduced glutathione (GsH; μmol g^−1^ FW) was estimated according to the method of Griffith (1980). The α-tocopherol was estimated in accordance with the methods of Konings et al. (1996) and Ching and Mohamed (2001). Finally, the level of hydrogen peroxide (H_2_O_2_ μmol g^−1^ leaf FW) was determined as reported by Velikova et al. (2000) and superoxide (${\text{O}}_{2}^{\cdot -}$) level was determined as described by Kubiś (2008).

### Determination of N, P, K^+^, and Ca^+2^ Content

Dried powder samples (0.1 g) were digested for 12 h in a mixture of 2 ml of perchloric acid (80%) with 10 ml of concentrated H_2_SO_4_. Each sample was diluted with distilled water to a volume of 100 ml. Total N was estimated using a micro-Kjeldahl method as described by Chapman and Pratt (1982). Total P was estimated colorimetrically using the ascorbic acid method as described by Watanabe and Olsen (1965). Flame photometry was used to analyze the K^+^ and Ca^+2^ content according to the method of Williams and Twine (1960).

### Preparation of Seed Extracts for Biochemical Analyses

The seeds were homogenized using a lab grinder and stored in airtight jars maintained at 4°C. Dried materials (10 g) were defatted by hexane 60–80 and then separately extracted successively with ethanol 70% (1:10 ratio) by soaking at room temperature for 12 h. The extract was centrifuged at 784 × *g* for 15 min (Jouan, MR 1822, Pays de la Loire, France). Extraction and filtration were repeated until the residue was colorless. The solvent was removed under vacuum at 40°C using a rotary evaporator (Laborota 4000-efficient, Heidolph, Germany), and extracts were freeze-dried using a lyophilizer. The obtained powder extracts were preserved in light-protected containers at −18°C.

### Determination of Biochemical Parameters

The antioxidant activity was determined using 2, 2-diphenyl-1-picrylhydrazyl (DPPH). The free radical scavenging activity was estimated as a percentage of DPPH discoloration as described by Blois (1958). The flavonoids content in the extracts was estimated following the colorimetric method based on the formation of flavonoid-aluminum compounds (Zhishen et al., 1999). Total polyphenols were estimated following the Folin–Ciocalteu method of Singleton and Rossi (1965).

### Sodium Dodecyl Sulfate-Polyacrylamide Gel Electrophoresis

Discontinuous Sodium dodecyl sulfate-polyacrylamide gel electrophoresis (SDS-PAGE) (Laemmli, 1970) was used to provide the molecular weight of the isolated proteins through comparisons to standard molecular weight markers (Marker, 28–250 kDa; Sigma-Aldrich). The protein bands were visualized by staining with Coomassie Brilliant Blue G-250 (Sigma-Aldrich).

### Statistical Analysis

The R statistical software version 3.6.1 was used to analyze the data. A combined analysis of variance was performed for the split-plot design with irrigation regimes, foliar applications, and their interactions as fixed effects, while growing seasons, replications, and their interactions were considered random effects. The differences among irrigation regimes, foliar applications, and their interactions were separated by the least significant difference at *p* ≤ 0.05. A biplot of a principal component analysis (PCA) estimated the association between evaluated traits.

## Results

### Agronomic Traits and CWP

**Figure 1:**
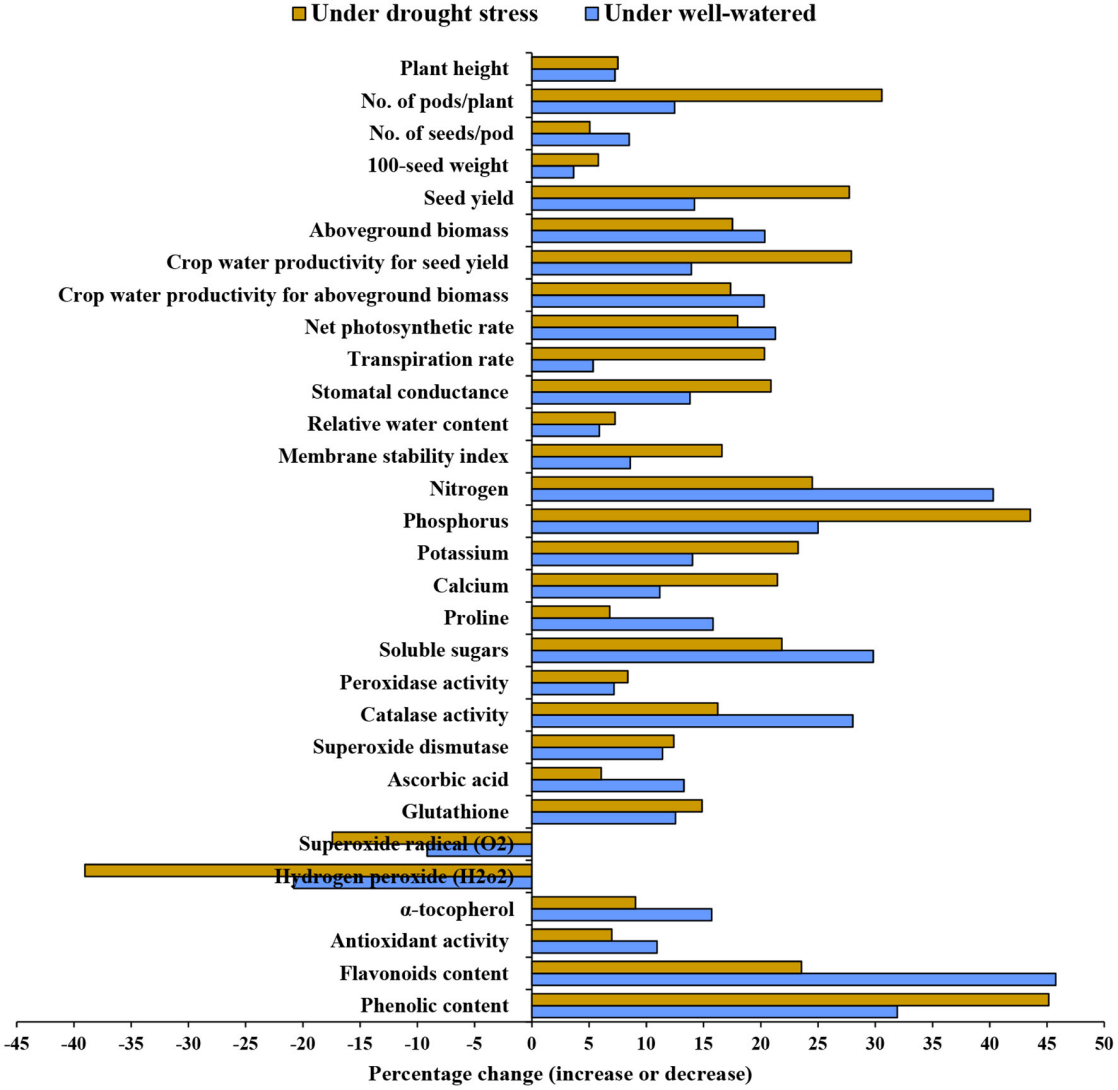
Percentage change (increase or decrease) in morphological, agronomic, physiological, and biochemical attributes upon the application of 1.5 mM nano-silicon dioxide (nano-SiO_2_) compared with untreated faba bean (*Vicia faba*) plants under well-watered and drought-stressed conditions.

The water-deficit treatment significantly decreased plant height, 100-seed weight, number of pods per plant, number of seeds per pod, seed yield, and aboveground biomass compared with the well-watered treatment (Table 1). Nano-SiO_2_ substantially enhanced all agronomic traits compared with the corresponding untreated plants under both watering regimes. The greatest yield was achieved with 1.5 mM, followed by 1 mM nano-SiO_2_ in the well-watered treatment. Treatment with 1.5 mM nano-SiO_2_ also increased the seed yield by 14.2% in well-watered plants and 27.7% in drought-stressed plants compared with those in untreated plants (Figure 1).

**Table 1:** Impacts of nano-silicon dioxide (nano-SiO_2_) foliar application on *Vicia faba* plant height (PH, cm), number of pods plant^−1^ (NP/P), number of seeds pod^−1^ (NS/P), 100-seed weight (100-SW, g), seed yield (SY, kg ha^−1^), aboveground biomass (AB, Kg ha^−1^), crop water productivity (kg m^−3^) for seed yield (CWP_s_), and aboveground biomass (CWP_ab_).

**Studied factor**		**PH**	**NP/P**	**NS/P**	**100-SW**	**SY**	**AB**	**CWP_**s**_**	**CWP_**ab**_**
**Irrigation**									
	Well-watered	116.7^a^	13.06^a^	3.07^a^	72.24^a^	5,362^a^	12,747^a^	1.31^b^	3.12^b^
	Droughted	107.7^b^	10.56^b^	2.85^b^	65.79^b^	4,221^b^	9,857^b^	1.50^a^	3.80^a^
**Foliar**									
	Control (tap water)	108.1^c^	10.76^d^	2.86^c^	67.89^b^	4,313^c^	10,192^c^	1.26^d^	2.99^d^
	0.5 mM nano-SiO_2_ (T1)	111.4^b^	11.36^c^	2.95^b^	68.62^b^	4,661^b^	11,077^b^	1.37^c^	3.26^c^
	1 mM nano-SiO_2_ (T2)	113.2^ab^	12.20^b^	3.01^ab^	69.98^a^	5,021^a^	11,798^a^	1.48^b^	3.45^b^
	1.5 mM nano-SiO_2_ (T3)	116.1^a^	12.93^a^	3.05^a^	71.07^a^	5,172^a^	12,140^a^	1.52^a^	3.55^a^
**Interaction**									
**Well-watered**	Control	112.1^c^	12.35^cd^	2.94^c^	70.91^c^	4,983^c^	11,473^c^	1.22^f^	2.81^g^
	T1	115.9^b^	12.79^bc^	3.05^b^	71.84^bc^	5,215^b^	12,261^b^	1.28^e^	3.00^f^
	T2	118.8^a^	13.19^b^	3.13^ab^	72.72^ab^	5,559^a^	13,443^a^	1.36^d^	3.29^e^
	T3	120.2^a^	13.89^a^	3.19^a^	73.50^a^	5,691^a^	13,808^a^	1.39^d^	3.38^d^
**Drought stress**	Control	104.2^e^	9.16^g^	2.77^d^	64.87^f^	3,643^f^	8,911^f^	1.29^e^	3.17^e^
	T1	106.9^de^	9.92^f^	2.85^cd^	65.40^f^	4,106^e^	9,892^e^	1.46^c^	3.52^c^
	T2	107.5^d^	11.21^e^	2.88^c^	67.24^e^	4,482^d^	10,153^de^	1.59^b^	3.61^b^
	T3	112.0^c^	11.96^d^	2.91^c^	68.64^d^	4,653^d^	10,472^d^	1.65^a^	3.72^a^
**ANOVA**	**DF**								
**Irrigation (I)**	1	0.0004	0.003	0.01	0.0005	0.002	0.001	0.0003	0.0001
**Foliar (F)**	3	0.009	<0.001	<0.001	0.0011	<0.001	<0.001	<0.001	<0.001
**I** **×** **F**	3	0.041	0.0001	0.01	0.003	0.047	0.002	<0.001	<0.001

*Means followed by the same letters under the same factor are not significantly different by the least significant difference at p ≤ 0.05*.

The CWP for seed yield (CWP_s_) ranged from 1.22 to 1.65 kg m^−3^, while the aboveground biomass (CWP_ab_) ranged from 2.81 to 3.72 kg m^−3^ (Table 1). Under drought stress conditions, faba bean plants possessed higher CWP_s_ (on average 1.5 kg m^−3^) and CWP_ab_ (3.8 kg m^−3^) than those that underwent the well-watered treatment (1.31 and 3.12 kg m^−3^, respectively). Drought-stressed plants also had a higher CWP than well-watered plants due to their more efficient water consumption and water loss reduction due to osmotic regulation. The application of nano-SiO_2_ also significantly improved CWP_s_ and CWP_ab_ compared with the untreated stressed controls. The highest CWP_s_ (1.65 kg m^−3^) and CWP_ab_ (3.72 kg m^−3^) were achieved with the application of 1.5 mM nano-SiO_2_.

### Physiological Parameters

Drought stress significantly reduced the net photosynthetic rate (*Pn*), transpiration rate (*Tr*), stomatal conductance (*gs*), RWC, MSI, and nutrient content (N, P, K^+^, and Ca^+2^) of faba bean plants (Table 2). All nano-SiO_2_ treatments mitigated the damage of drought stress and significantly improved all physiological parameters. The highest values were seen in well-watered plants treated with 1.5 mM nano-SiO_2_, while the lowest values were recorded in the control water-stressed plants (Table 2).

**Table 2:** Impacts of nano-silicon dioxide (nano-SiO_2_) foliar application on the net photosynthetic rate (*Pn*, μmol CO_2_ m^−2^ s^−1^), transpiration rate (*Tr*, μmol CO_2_ m^−2^ s^−1^), stomatal conductance (*gs*, μmol CO_2_ m^−2^ s^−1^), relative water content (RWC, %), membrane stability index (MSI, %), nitrogen (N, %), phosphorus (P, %), potassium (K, %), and calcium (Ca, %) contents of faba bean (*Vicia faba*) grown under well-watered and drought-stressed conditions over two growing seasons (2019 and 2020).

**Studied factor**		***Pn***	***Tr***	***gs***	**RWC**	**MSI**	**N**	**P**	**K**	**Ca**
**Irrigation**										
	Well-watered	12.23^a^	7.05^a^	0.54^a^	77.01^a^	60.94^a^	2.37^a^	0.35^a^	2.25^a^	2.30^a^
	Droughted	8.44^b^	4.41^b^	0.34^b^	59.72^b^	35.40^b^	1.62^b^	0.18^b^	1.28^b^	1.32^b^
**Foliar**										
	Control (tap water)	9.44^d^	5.42^d^	0.41^d^	66.19^d^	45.58^d^	1.72^d^	0.20^d^	1.62^d^	1.69^d^
	0.5 mM nano-SiO_2_ (T1)	10.09^c^	5.64^c^	0.43^c^	67.76^c^	47.34^c^	1.89^c^	0.26^c^	1.74^c^	1.77^c^
	1 mM nano-SiO_2_ (T2)	10.49^b^	5.85^b^	0.45^b^	69.02^b^	48.95^b^	2.10^b^	0.28^b^	1.81^b^	1.85^b^
	1.5 mM nano-SiO_2_ (T3)	11.32^a^	6.01^a^	0.47^a^	70.49^a^	50.81^a^	2.29^a^	0.30^a^	1.90^a^	1.94^a^
**Interaction**										
**Well-watered**	Control	11.12^c^	6.86^d^	0.51^d^	74.58^d^	58.42^d^	1.98^d^	0.30^c^	2.09^c^	2.18^d^
	T1	11.99^b^	7.00^c^	0.53^c^	76.64^c^	60.24^c^	2.21^c^	0.34^b^	2.23^b^	2.27^c^
	T2	12.32^b^	7.13^b^	0.55^b^	77.84^b^	61.64^b^	2.53^b^	0.36^a^	2.29^b^	2.33^b^
	T3	13.49^a^	7.23^a^	0.58^a^	78.98^a^	63.44^a^	2.77^a^	0.38^a^	2.38^a^	2.42^a^
**Drought stress**	Control	7.75^g^	3.99^h^	0.30^h^	57.80^h^	32.73^h^	1.46^f^	0.10^f^	1.15^f^	1.20^h^
	T1	8.19^f^	4.28^g^	0.33^g^	58.87^g^	34.43^g^	1.56^ef^	0.19^e^	1.25^e^	1.28^g^
	T2	8.65^e^	4.56^f^	0.35^f^	60.20^f^	36.25^f^	1.67^e^	0.20^e^	1.32^e^	1.36^f^
	T3	9.15^d^	4.80^e^	0.37^e^	62.00^e^	38.17^e^	1.81^d^	0.22^d^	1.41^d^	1.45^e^
**ANOVA**	**DF**									
**Irrigation (I)**	1	0.0004	<0.001	0.0001	0.0003	0.0001	0.002	0.0042	0.001	0.0006
**Foliar (F)**	3	<0.001	<0.001	<0.001	<0.001	<0.001	<0.001	<0.001	<0.001	<0.001
**I** **×** **F**	3	0.018	0.0001	0.036	0.045	0.047	0.0003	0.005	0.037	0.039

*Means followed by the same letters under the same factor are not significantly different by least significant difference at p ≤ 0.05*.

Drought-stressed plants increased free proline and soluble sugars and the activity of the antioxidant enzymes CAT, POD, and SOD. Furthermore, the non-enzymatic antioxidants AsA, GsH, and α-tocopherol were higher compared with non-stressed faba beans (Table 3). In contrast, the application of nano-SiO_2_ significantly increased the free proline and soluble sugar contents and the activities of antioxidant enzymes and non-enzymatic antioxidant compounds compared with the untreated plants under both irrigation regimes. The highest values were achieved with the application of 1.5 mM of nano-SiO_2_ to the stressed plants (Table 3). Increasing free proline, soluble sugars, antioxidant enzyme activities, and non-enzymatic antioxidant compound content helped the faba bean plants alleviate the negative impacts of water scarcity.

**Table 3:** Impacts of nano-silicon dioxide (nano-SiO_2_) foliar application on the proline content (Pro, μmol g^−1^ DW), soluble sugars (SSu, mg g^−1^ DW), peroxidase activity (POD, unit mg^−1^ protein), catalase activity (CAT, unit mg^−1^ protein), superoxide dismutase activity (SOD, unit mg^−1^ protein), ascorbic acid (AsA, μmol g^−1^ DW), glutathione (GsH, μmol g^−1^ FW), superoxide radical (${\text{O}}_{2}^{\cdot -}$, A580 g^−1^ FW), hydrogen peroxide (H_2_O_2_, μmol g^−1^ FW), and α-tocopherol (α-TOC, μmol g^−1^ DW) of well-watered and drought-stressed faba beans (*Vicia faba*) over two growing seasons (2019 and 2020).

**Studied factor**		**Pro**	**SSu**	**POD**	**CAT**	**SOD**	**AsA**	**GsH**	** ${\text{O}}_{2}^{\cdot -}$ **	**H_**2**_O_**2**_**	**α-TOC**
**Irrigation**											
	Well-watered	66.76^b^	23.38^b^	78.20^b^	0.50^b^	42.22^b^	1.36^b^	0.33^b^	0.41^b^	4.28^b^	1.58^b^
	Droughted	177.4^a^	53.40^a^	137.5^a^	0.85^a^	75.45^a^	2.94^a^	0.49^a^	0.59^a^	12.14^a^	3.63^a^
**Foliar**											
	Control (tap water)	116.5^d^	33.92^d^	103.8^d^	0.61^d^	55.51^d^	2.07^d^	0.39^c^	0.55^a^	10.55^a^	2.45^c^
	0.5 mM nano-SiO_2_ (T1)	121.4^c^	36.91^c^	105.8^c^	0.65^c^	57.75^c^	2.11^c^	0.41^b^	0.51^b^	8.19^b^	2.59^b^
	1 mM nano-SiO_2_ (T2)	123.2^b^	40.58^b^	109.7^b^	0.69^b^	59.89^b^	2.17^b^	0.41^b^	0.48^c^	7.39^c^	2.65^b^
	1.5 mM nano-SiO_2_ (T3)	127.2^a^	42.13^a^	112.0^a^	0.74^a^	62.19^a^	2.24^a^	0.44^a^	0.46^d^	6.71^d^	2.72^a^
**Interaction**											
**Well-watered**	Control	61.47^g^	20.23^f^	75.21^f^	0.44^h^	39.91^g^	1.28^f^	0.31^d^	0.44^e^	5.04^e^	1.44^f^
	T1	66.99^f^	22.20^e^	77.77^e^	0.47^g^	41.47^fg^	1.33^f^	0.33^c^	0.42^e^	4.45^f^	1.57^e^
	T2	67.35^f^	24.80^d^	79.22^de^	0.51^f^	43.03^ef^	1.38^e^	0.34^c^	0.39^f^	3.98^fg^	1.63^de^
	T3	71.21^e^	26.27^d^	80.60^d^	0.56^e^	44.46^e^	1.45^d^	0.35^c^	0.37^f^	3.64^g^	1.67^d^
**Drought stress**	Control	171.5^d^	47.61^c^	132.4^c^	0.78^d^	71.11^d^	2.86^c^	0.47^b^	0.65^a^	16.06^a^	3.46^c^
	T1	175.9^c^	51.61^b^	133.9^c^	0.83^c^	74.02^c^	2.90^c^	0.48^b^	0.59^b^	11.93^b^	3.61^b^
	T2	179.1^b^	56.37^a^	140.3^b^	0.86^b^	76.74^b^	2.96^b^	0.49^b^	0.57^c^	10.79^c^	3.67^b^
	T3	183.1^a^	58.00^a^	143.5^a^	0.91^a^	79.92^a^	3.03^a^	0.54^a^	0.54^d^	9.79^d^	3.77^a^
**ANOVA**	**DF**										
**Irrigation (I)**	1	0.0001	0.0003	<0.001	0.0001	0.0002	0.0006	0.0013	0.0008	0.0003	0.006
**Foliar (F)**	3	<0.001	<0.001	<0.001	<0.001	<0.001	<0.001	<0.001	<0.001	<0.001	<0.001
**I** **×** **F**	3	0.024	0.009	0.0004	0.048	0.009	0.049	0.007	0.014	<0.001	0.045

*Means followed by the same letters under the same factor are not significantly different by least significant difference at p ≤ 0.05*.

In addition, irrigation regimes and nano-SiO_2_ application significantly impacted oxidative stress biomarkers (Table 3). The levels of H_2_O_2_ and ${\text{O}}_{2}^{\cdot -}$ were significantly increased in stressed compared with non-stressed plants. Furthermore, the application of nano-SiO_2_ to plants in the water-deficit treatment significantly lowered H_2_O_2_ and ${\text{O}}_{2}^{\cdot -}$ levels compared with those in untreated plants.

### Biochemical Parameters

Drought stress caused a significant increase in antioxidant activity, flavonoids, and phenolics (Table 4). The application of nano-SiO_2_ significantly enhanced these parameters compared with those in the untreated plants under both irrigation regimes. The highest antioxidant activity and values of flavonoids and phenolics were produced by the 1.5-mM nano-SiO_2_ application in the water-deficit treatments, while the lowest values were recorded in the well-watered control plants (Table 4).

**Table 4:** Impact of nano-silicon dioxide (nano-SiO_2_) foliar application on antioxidant activity (%), flavonoids content (μg QE/g), and phenolic content (μg GAE/g) of faba beans (*Vicia faba*) under well-watered or drought-stressed conditions.

**Studied factor**		**Antioxidant activity (%)**	**Flavonoids content (μg QE/g extract)**	**Phenolic content (μg GAE/g extract)**
**Irrigation**				
	Well-watered	81.43^b^	106.7^b^	1,467^b^
	Droughted	89.50^a^	134.8^a^	2,182^a^
**Foliar**				
	Control (tap water)	82.06^d^	103.5^b^	1,482^d^
	0.5 mM nano-SiO_2_ (T1)	84.24^c^	113.4^b^	1,805^c^
	1 mM nano-SiO_2_ (T2)	86.24^b^	128.5^a^	1,945^b^
	1.5 mM nano-SiO_2_ (T3)	89.31^a^	137.6^a^	2,066^a^
**Interaction**				
**Well-watered**	Control	77.03^f^	87.68^d^	1,275^g^
	T1	80.10^e^	95.90^d^	1,398^f^
	T2	83.13^d^	115.3^cd^	1,512^e^
	T3	85.45^c^	127.8^bc^	1,682^d^
**Drought stress**	Control	87.09^c^	119.3^bcd^	1,688^d^
	T1	88.38^bc^	130.9^bc^	2,211^c^
	T2	89.35^b^	141.7^b^	2,377^b^
	T3	93.17^a^	147.4^a^	2,450^a^
**ANOVA**	**DF**			
**Irrigation (I)**	1	0.013	0.039	<0.001
**Foliar (F)**	3	<0.001	0.019	<0.001
**I** **×** **F**	3	0.003	0.015	<0.001

*Means followed by the same letters under the same factor are not significantly different by least significant difference at p ≤ 0.05*.

*QE, quercetin; GAE, galic acid*.

### Sodium Dodecyl Sulfate-Polyacrylamide Gel Electrophoresis Protein Pattern

The SDS-PAGE patterns displayed nine protein bands (170, 158, 110, 90, 81, 63, 57, 40, and 30 kD) in untreated faba bean plants grown under well-watered or water-deficit conditions (Table 5). However, the nano-SiO_2_ concentrations of 1 and 1.5 mM created a protein fraction with a molecular weight of 129 kD only in the faba bean plants grown under drought stress. Only nine bands were observed in the rest of the treatments in both watering regimes.

**Table 5:** Effect of nano-silicon dioxide (nano-SiO_2_) on protein profile in faba bean (*Vicia faba*) seeds under well-watered and drought-stressed conditions.

**Band No**.	**Molecular weight (KD)**	**Retention factor**	**Well-watered**				**Drought-stressed**			
			**Tap water**	**0.5 mM n-SiO_**2**_**	**1 mM n-SiO_**2**_**	**1.5 mM n-SiO_**2**_**	**Tap water**	**0.5 mM n-SiO_**2**_**	**1 mM n-SiO_**2**_**	**1.5 mM n-SiO_**2**_**
1	170	0.066	+	+	+	+	+	+	+	+
2	158	0.101	+	+	+	+	+	+	+	+
3	129	0.189	–	–	–	–	–	–	+	+
4	110	0.260	+	+	+	+	+	+	+	+
5	90	0.348	+	+	+	+	+	+	+	+
6	81	0.395	+	+	+	+	+	+	+	+
7	63	0.507	+	+	+	+	+	+	+	+
8	57	0.551	+	+	+	–	+	+	+	+
9	40	0.699	+	+	+	+	+	+	+	+
10	30	0.847	+	+	+	+	+	+	+	+

### Interrelationship Among Evaluated Traits

**Figure 2:**
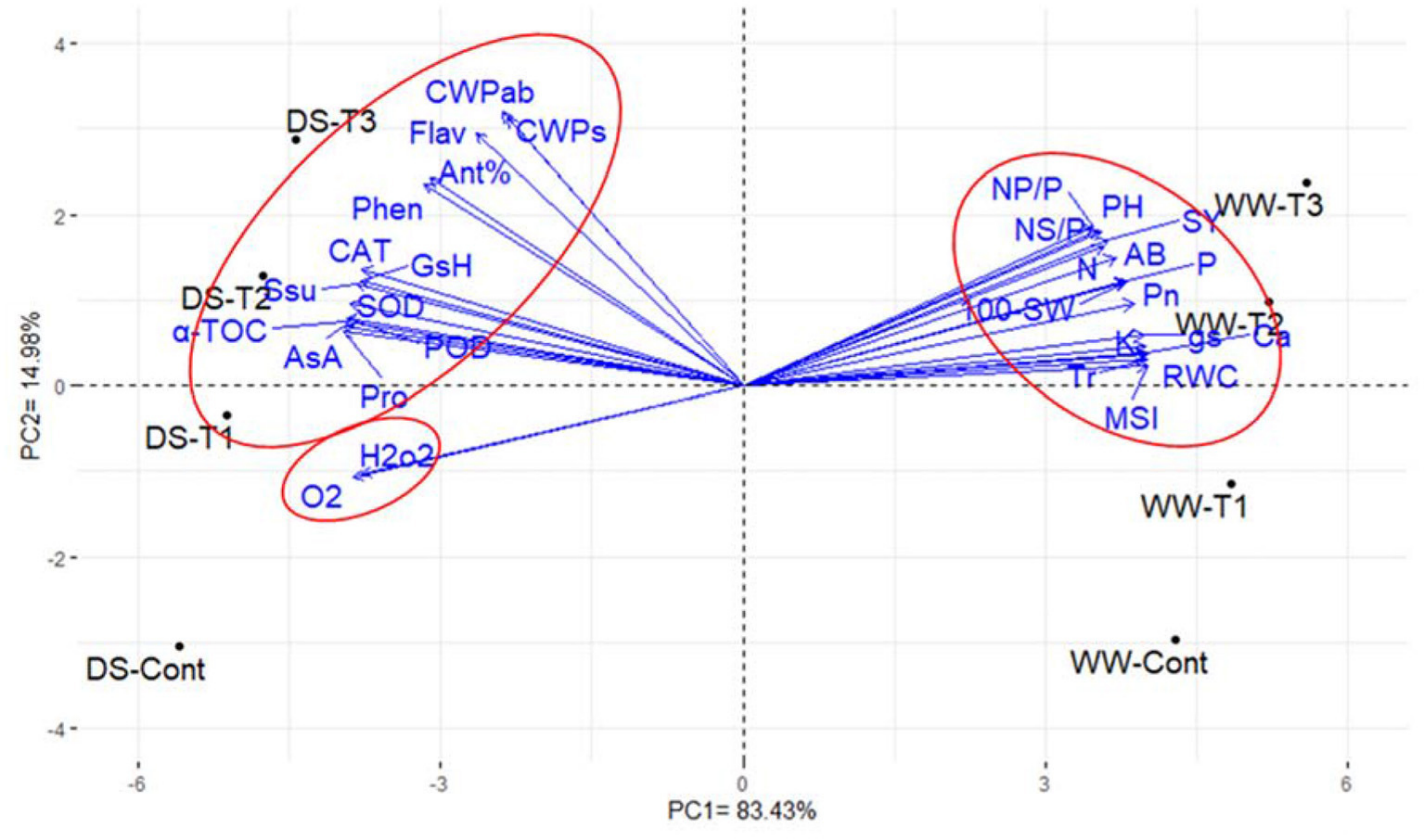
Biplot of the first two principal components for the physiological, biochemical, and agronomic traits of faba beans (*Vicia faba*). The physiological parameters comprised of net photosynthetic rate (*Pn*), transpiration rate (*Tr*), stomatal conductance (*gs*), relative water content (RWC), membrane stability index (MSI), nitrogen (N), phosphorus (P), potassium (K), calcium (Ca) content, proline content (Pro), soluble sugars (SSu), peroxidase activity (POD), catalase activity (CAT), superoxide dismutase activity (SOD), ascorbic acid (AsA), glutathione (GsH), hydrogen peroxide (H_2_O_2_), superoxide radical (${\text{O}}_{2}^{.-}$), and α-tocopherol (α-TOC). The biochemical parameters included antioxidant activity % (Ant%), flavonoids content (Flav), and phenolic content (Phen). The agronomic traits were plant height (PH), number of pods plant^−1^ (NP/P), number of seeds pod^−1^ (NS/P), 100-seed weight (100-SW), seed yield (SY), aboveground biomass (AB), crop water productivity for seed yield (CWPs), and aboveground biomass (CWPab). WW-Cont, WW-T1, WW-T2, and WW-T3 were foliar applications using tap water, 0.5, 1, and 1.5 mM of nano-silicon dioxide (nano-SiO_2_) under the well-watered treatment, respectively. DS-Cont, DS-T1, DS-T2, and DS-T3 were foliar applications using tap water, 0.5, 1, and 1.5 mM of nano-SiO_2_ under the drought stress treatment.

The PCA of the agronomic, physiological, and biochemical traits showed that the first two principal components accounted for most of the variability, which was ~98.41% (83.43% by PC1 and 14.98% by PC2; Figure 2). In the PC biplot, the traits were represented by parallel vectors or those that were close to each other, indicating a strong positive association, whereas those that were situated approximately opposite (at 180°) showed a highly negative relationship. In addition, the vectors toward the sides expressed a slight relationship. The 30 investigated traits could be classified into three groups: the first group (15 traits) comprises agronomic traits, namely, *Pn, Tr, gs*, MSI, RWC, and nutrient contents (N, P, K^+^, and Ca^+2^), the second group (13 traits) consisted of the CWP for seed yield and aboveground biomass, antioxidant activity, flavonoids and phenolic contents, proline and soluble sugar contents, enzymatic antioxidants (POD, CAT, and SOD), and non-enzymatic antioxidant compounds (AsA, GsH, α-TOC, proline, and soluble sugars), and the third group contained H_2_O_2_ and ${\text{O}}_{2}^{.-}$. Furthermore, PC1 separated the treatments into two groups. The well-watered treatments were located on the positive side, while those under drought stress were located on the negative side (Figure 2). The traits in the first group were associated with well-watered plants, especially those that were treated with 1 and 1.5 mM nano-SiO_2_, while the biochemical attributes in the second and third groups were associated with the drought-stressed treatments.

## Discussion

Water scarcity stress poses great challenges to sustainable faba bean production due to its sensitivity to drought. Consequently, it is vital to identify novel approaches to enhance drought tolerance, especially under conditions of abrupt climate change. In the current study, drought-stressed faba bean plants exhibited a decline in seed yield-related traits and CWP and an increase in oxidative and osmotic stress compared with those of well-watered plants. As a result, exogenously applied nano-SiO_2_ enhanced gas exchange, water relations, nutrient uptake, activities of antioxidant enzymes, and non-enzymatic antioxidant compounds, which substantially reduced oxidative stress and positively reflected the improvement of drought tolerance in faba bean plants with enhancements in yield-related traits and CWP.

The Si nanoparticles have great availability and are easily absorbed by plants compared with bulk Si, consequently supporting greater ameliorative impacts under abiotic stresses (Suriyaprabha et al., 2012; Tripathi et al., 2016). The obtained results demonstrated that the application of nano-SiO_2_, particularly at 1.5 mM, considerably boosted photosynthetic effectiveness and leaf gas exchange (e.g., *Pn, Tr*, and *gs*) in plants under water-deficit stress compared to those that were untreated. Promoting photosynthetic efficiency maintenance could be attributed to optimal stomatal conductance and strong antioxidant activities, which improve plant tolerance to drought stress (Suriyaprabha et al., 2012; Haghighi and Pessarakli, 2013; Rios et al., 2017; Jia et al., 2021). Furthermore, the application of nano-SiO_2_ increased the water uptake from roots to leaves by enhancing the RWC and MSI in treated faba bean plants compared with those in control plants under drought stress. Previous studies have also found that maintaining RWC and MSI at a healthy status enhances osmotic adjustments and metabolic activities under conditions of drought stress (Slabbert and Krüger, 2014). In this study, nano-SiO_2_ application likely boosted the water content of plants by decreasing stomatal transpiration and increasing turgor pressure and potassium absorbance (Romero-Aranda et al., 2006; Tahir et al., 2012; Liu et al., 2014; Cerný et al., 2020). Furthermore, nano-SiO_2_ application significantly improved the absorption of the nutrients N, P, K^+^, and Ca^+2^ in faba bean plants under drought stress. In addition, it has been found that mineral nutrient uptake depends mainly on membrane activity, which plays a fundamental role in the ion movement from the soil to the plant and controls the allocation of cells (Khoshgoftarmanesh et al., 2014; Gupta et al., 2016). These enhancements in water movement and nutrient absorption enable improvements in all physiological activities of the cells.

Within cells, the accumulation of compatible solutes under stress effectively maintains the status of plant cell water. For example, proline is an excellent compatible osmolyte that improves plant antioxidant systems and affects osmotic adjustment (Zhu, 2001; Desoky et al., 2020). Soluble sugars also maintain the balance between the vacuole and the osmotic quality of cytosol under abiotic stress conditions (Sairam et al., 2002). The results indicated that the application of nano-SiO_2_ elevated the proline and soluble sugar contents in drought-stressed plants. Therefore, its application improved plant tolerance by elevating osmolyte content, altering osmotic potential, and maintaining higher turgor in response to drought stress (Claussen, 2005; Rizwan et al., 2015; Zhang et al., 2017; Desoky et al., 2020). Furthermore, improved enzymatic and non-enzymatic defense systems can alleviate the overproduction of ROS, such as ${\text{O}}_{2}^{\cdot -}$ and H_2_O_2_, produced in response to drought stress. The enzymatic defense system components SOD, CAT, and POD and the non-enzymatic defense system components GsH, AsA, and α-TOC were significantly increased following the application of nano-SiO_2_ compared with those in untreated plants under drought stress. Accordingly, the application of nano-SiO_2_ lowered the concentrations of ${\text{O}}_{2}^{\cdot -}$ and H_2_O_2_ and, therefore, reduced oxidative stress in drought-stressed faba bean plants. Consequently, the application of nano-SiO_2_ could be an effective tool to increase faba bean tolerance to oxidative stress by enhancing ROS scavenging enzymatic and non-enzymatic defense systems (Rios et al., 2017; Rady et al., 2019).

The application of nano-SiO_2_ enhanced flavonoids, phenolic compounds, and antioxidant activity levels, particularly in drought-stressed faba bean plants. The utilization of these protection systems helps to overcome oxidative damage, which has been suggested to occur through the synthesis of secondary metabolites, such as flavonoids and phenolic contents (Blokhina et al., 2003). These metabolites can forestall protein denaturation, DNA damage, and lipid peroxidation (Król et al., 2014; Quan et al., 2016). Although Si is not a fundamental component of plants, it has a beneficial impact in improving protection against drought stress through the initiation of protective proteins and the reduction of ROS (Suriyaprabha et al., 2013; Luyckx et al., 2017). In addition, the electrograph of the protein fraction profile showed that the application of 1 and 1.5 mM nano-SiO_2_ led to the generation of a new protein fraction (molecular weight: 129 kD).

The application of nano-SiO_2_ exhibited significant positive alterations in all the investigated physiological and biochemical attributes under water-deficit conditions. Accordingly, treated plants grew more efficiently under drought stress compared with untreated plants and coped with water deficit conditions. Nano-SiO_2_ application, particularly using a concentration of 1.5 mM as an optimum level, attenuated the devastating impacts of drought stress by improving photosynthetic efficiency, plant water status, nutrient absorption, nutrient uptake, non-enzymatic and enzymatic antioxidant activities, and ROS scavenging. These promotional influences were reflected in increasing seed yield and all its related traits and CWP compared with the corresponding untreated plants.

The interrelationship among the evaluated parameters (Figure 2) reflected that the agronomic traits positively associated with *Pn, Tr, gs*, MSI, RWC, and nutrient contents (physiological parameters). We speculate that the high values of these physiological parameters are associated with greater seed yield and contributing traits, particularly under conditions of drought stress. Otherwise, the CWP_s_ and CWP_ab_ demonstrated a highly positive association with antioxidant activity, flavonoids, and phenolic compounds. In addition, the agronomic traits exhibited a highly negative association with H_2_O_2_ and ${\text{O}}_{2}^{.-}$ (Desoky et al., 2020; Mansour et al., 2020, 2021). According to these results, it is interesting to detect specific physiological and biochemical parameters highly associated with yield-related traits or CWP under water-deficit conditions.

## Conclusion

Drought stress in faba bean plants resulted in reduced gas exchange, leaf water potential, leaf turgidity, nutrient uptake, photosynthetic rate, and flavonoids and phenolic content compared with the same levels in well-watered plants. These adverse impacts were reflected in decreased yield-related traits and CWP. However, exogenously applied nano-SiO_2_, particularly at 1.5 mM, mitigated the negative impacts of drought stress and promoted plant growth by boosting all of the investigated physiological attributes. In addition, the application of nano-SiO_2_ decreased the membrane leakage of electrolytes and membrane lipid peroxidation due to reduced drought-stimulated oxidative stress by enhancing the activity of osmoprotectants and the enzymatic and non-enzymatic defense system components under drought stress. Consequently, nano-SiO_2_ had a positive influence on the physiological, biochemical, and agronomic traits of drought-stressed plants and alleviated the influence of drought stress on faba bean plants.

## Data Availability Statement

The original contributions presented in the study are included in the article/Supplementary Material, further inquiries can be directed to the corresponding author.

## Author Contributions

ED, EM, EAE, MA, TT, HE, MY, and KE-T conceived and designed the experiments. SA and RE helped in conducting the experiments and collected the literature. ED, MA, TT, HE, and KE-T analyzed the data and drafted the manuscript. EM, EAE, RE, MY, and KE-T wrote and made the final edits to the manuscript. All the authors read and approved the final version of the manuscript.

## Conflict of Interest

The authors declare that the research was conducted in the absence of any commercial or financial relationships that could be construed as a potential conflict of interest.

## Publisher's Note

All claims expressed in this article are solely those of the authors and do not necessarily represent those of their affiliated organizations, or those of the publisher, the editors and the reviewers. Any product that may be evaluated in this article, or claim that may be made by its manufacturer, is not guaranteed or endorsed by the publisher.
