# Supplementary material for: Physio-Biochemical and Agronomic Responses of Faba Beans to Exogenously Applied Nano-Silicon Under Drought Stress Conditions
Source: Front Plant Sci. 2021 Sep 16;12:637783. doi: 10.3389/fpls.2021.637783 (PMC8481644; doi:10.3389/fpls.2021.637783)
Supplement: Supplementary file 1 [file Data_Sheet_1.pdf]

# Supplementary Materials

# Physio-Biochemical and Agronomic Responses of Faba Beans to Exogenously Applied Nano-Silicon under Drought Stress Conditions

***El-Sayed M. Desoky, Elsayed Mansour, El-Sayed E. A. El-Sobky, Mohamed I. Abdul-Hamid, Taha F. Taha, Hend A. Elakkad, Safaa M.A.I Arnaout, Rania S. M. Eid, Khaled A. El-Tarabily*, Mohamed A.T. Yasin***

*** Correspondence:**

Khaled El-Tarabily: ktarabily@uaeu.ac.ae

**Supplementary Table 1.** Soil properties at the experimental site.

**Supplementary Table 2.** Monthly minimum (Tmin, ˚C) and maximum (Tmax, ˚C) temperatures and precipitation (Prec., mm).

**Supplementary Figure 1.** Characterization of chemical silicon (Si) nanoparticles (SiNPs); (A) maximum UV absorbance at 260 nm, (B) spherical shape by transmission electron microscopy (TEM), (C, D) size (51.35 nm) and charge (-21.28) of SiNPs by zeta seizer, and zeta potential, respectively, and (E) X-ray diffraction (XRD) was used to identify the crystalline nature of SiNPs.

**Supplementary Figure 2.** Soluble sugars standard curve at 520 nm absorbance.

**Supplementary Figure 3.** Proline standard curve at 520 nm absorbance.

**Supplementary Table 1.** Soil properties at the experimental site.

| Soil depth (cm) | Soil bulk density (g cm $^{-3}$) | Field capacity (%) | Wilting point (%) | pH | Sand (%) | Silt (%) | Clay (%) | Texture |
|---|---|---|---|---|---|---|---|---|
| **0-30** | 1.49 | 7.25 | 1.65 | 8.05 | 94.95 | 3.33 | 1.72 | Sandy |
| **30-60** | 1.56 | 8.68 | 1.71 | 7.91 | 95.19 | 3.21 | 1.60 | Sandy |
| **60-90** | 1.61 | 8.11 | 1.55 | 7.80 | 96.33 | 2.54 | 1.13 | Sandy |

**Supplementary Table 2.** Monthly minimum (Tmin, ˚C) and maximum (Tmax, ˚C) temperatures and precipitation (Prec., mm).

| Month | 2018-2019 | | | 2019-2020 | | | 35-year average (1986-2020) | | |
|---|---|---|---|---|---|---|---|---|---|
| | Tmin | Tmax | Prec. | Tmin | Tmax | Prec. | Tmin | Tmax | Prec. |
| **November** | 14.4 | 26.9 | 5.7 | 14.9 | 28.5 | 5.9 | 13.6 | 25.6 | 9.7 |
| **December** | 10.9 | 21.2 | 9.8 | 9.6 | 21.0 | 9.2 | 9.8 | 21.1 | 7.1 |
| **January** | 7.0 | 19.1 | 10.3 | 7.3 | 18.0 | 9.6 | 8.2 | 19.5 | 11.0 |
| **February** | 9.0 | 20.1 | 12.9 | 8.1 | 20.5 | 15.1 | 9.1 | 20.7 | 10.3 |
| **March** | 11.3 | 23.0 | 11.2 | 10.1 | 24.8 | 15.6 | 11.3 | 23.7 | 8.1 |
| **April** | 13.8 | 26.2 | 3.2 | 12.4 | 25.3 | 4.1 | 14.2 | 28.0 | 4.8 |

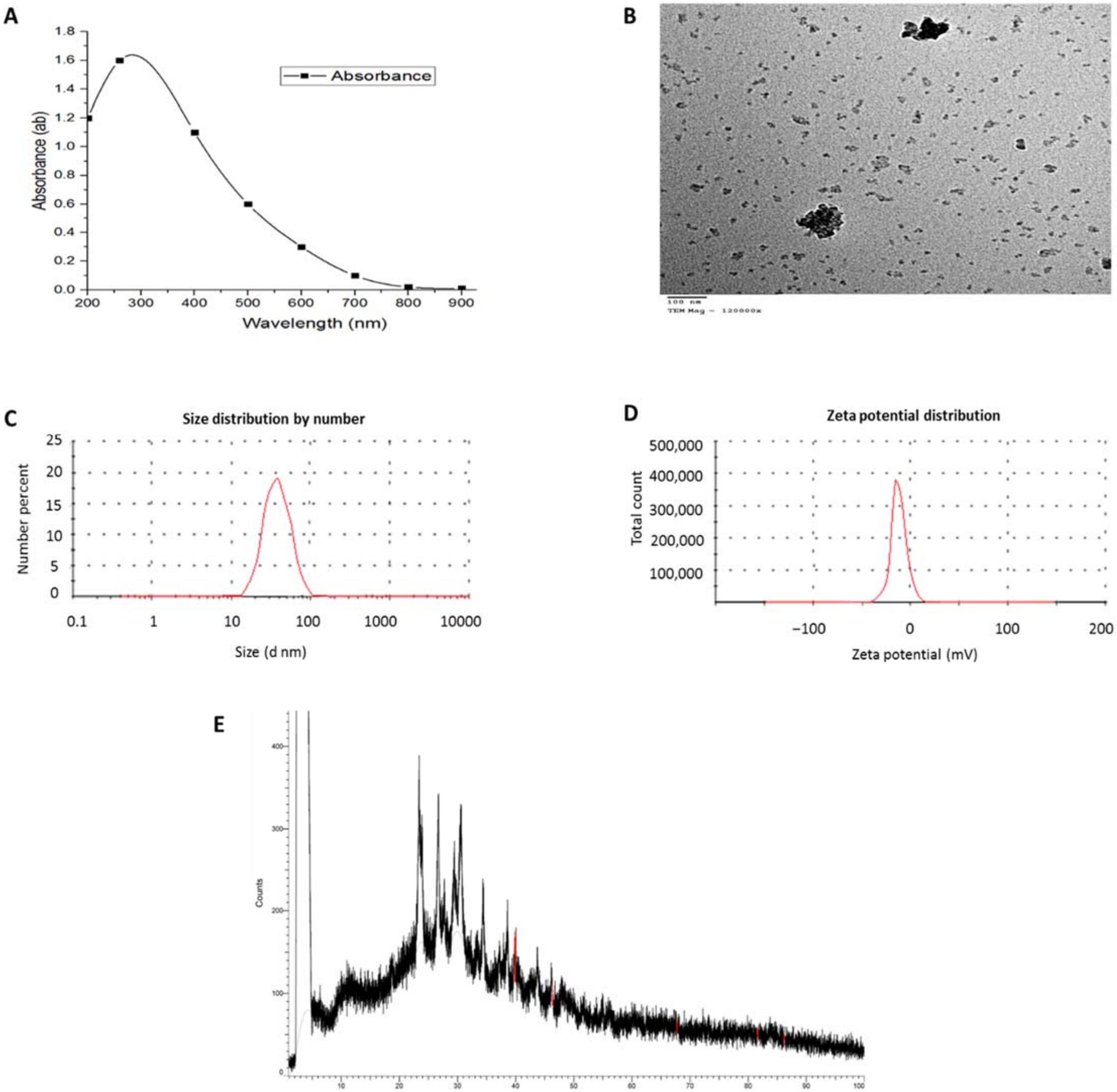


**Supplementary Figure 1.** Characterization of chemical silicon (Si) nanoparticles (SiNPs); (A) maximum UV absorbance at 260 nm, (B) spherical shape by transmission electron microscopy (TEM), (C, D) size (51.35 nm) and charge (-21.28) of SiNPs by zeta seizer, and zeta potential, respectively, and (E) X-ray diffraction (XRD) was used to identify the crystalline nature of SiNPs.

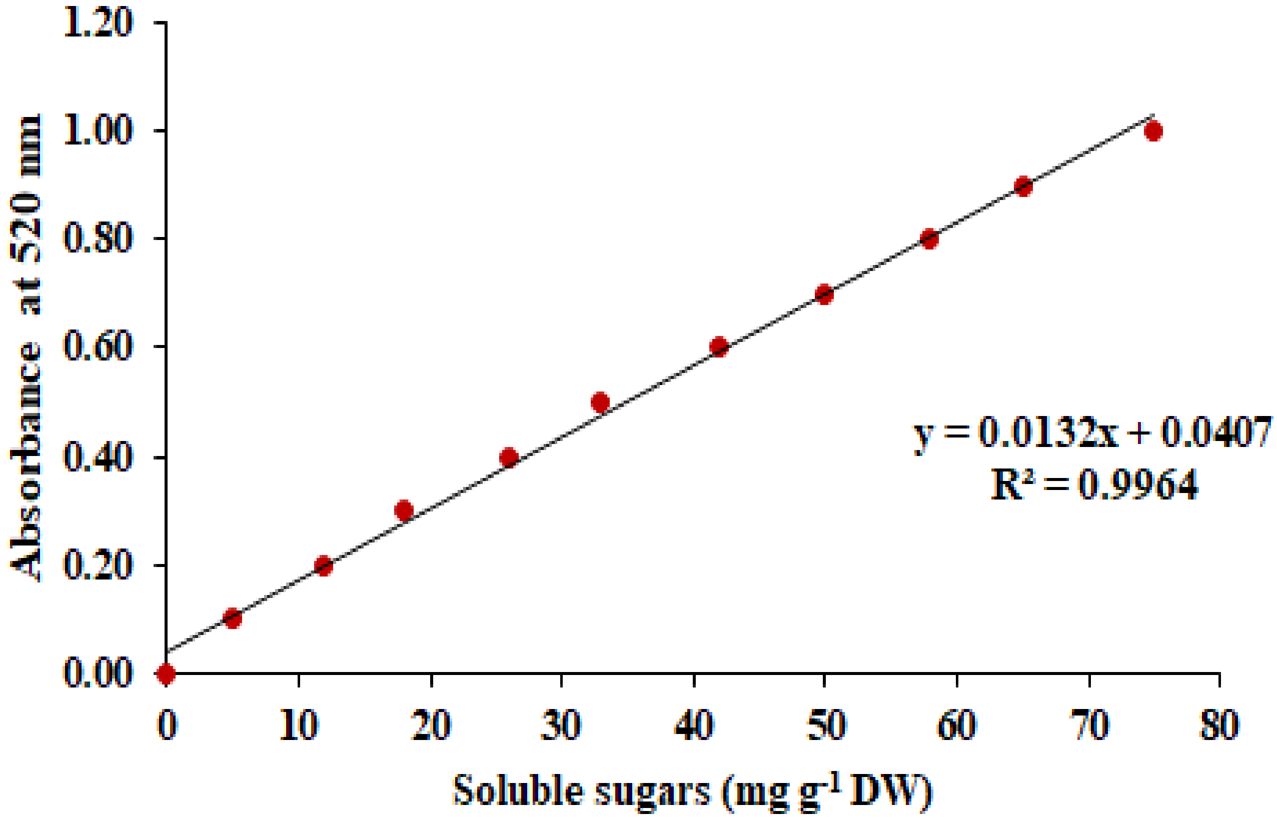


**Supplementary Figure 2.** Soluble sugars standard curve at 520 nm absorbance.

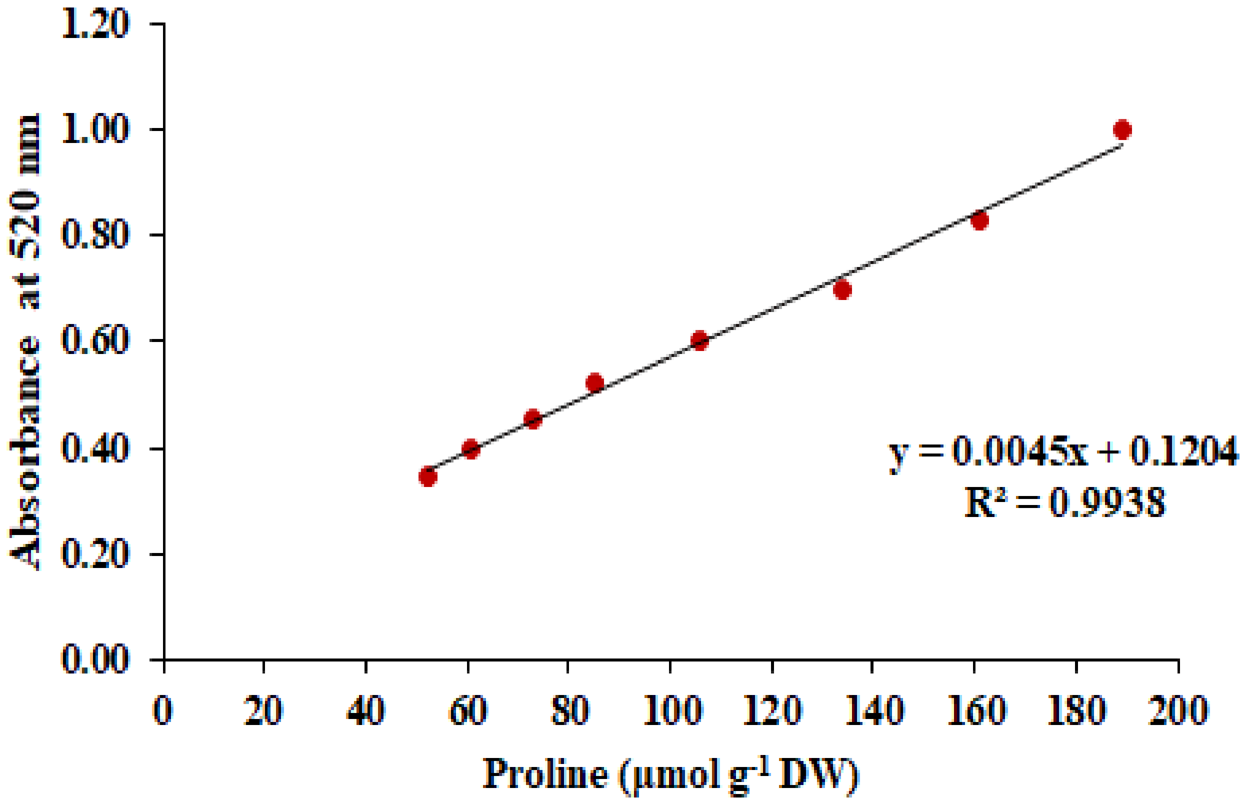


**Supplementary Figure 3.** Proline standard curve at 520 nm absorbance.
